# Chromium inhibition and size-selected Au nanocluster catalysis for the solution growth of low-density ZnO nanowires

**DOI:** 10.1038/srep12336

**Published:** 2015-07-23

**Authors:** Vito Errico, Giuseppe Arrabito, Simon R. Plant, Pier Gianni Medaglia, Richard E. Palmer, Christian Falconi

**Affiliations:** 1 Department of Electronic Engineering, University of Rome Tor Vergata, Via del Politecnico 1, 00133, Rome, Italy; 2 Nanoscale Physics Research Laboratory, School of Physics and Astronomy, University of Birmingham, Edgbaston, Birmingham, B15 2TT, United Kingdom; 3 Department of Industrial Engineering, University of Rome Tor Vergata, Via del Politecnico 1, 00133, Rome, Italy

## Abstract

The wet chemical synthesis of nanostructures has many crucial advantages over high-temperature methods, including simplicity, low-cost, and deposition on almost arbitrary substrates. Nevertheless, the density-controlled solution growth of nanowires still remains a challenge, especially at the low densities (*e.g.* 1 to 10 nanowires/100 μm^2^) required, as an example, for intracellular analyses. Here, we demonstrate the solution-growth of ZnO nanowires using a thin chromium film as a nucleation inhibitor and Au size-selected nanoclusters (SSNCs) as catalytic particles for which the density and, in contrast with previous reports, size can be accurately controlled. Our results also provide evidence that the enhanced ZnO hetero-nucleation is dominated by Au SSNCs catalysis rather than by layer adaptation. The proposed approach only uses low temperatures (≤70 °C) and is therefore suitable for any substrate, including printed circuit boards (PCBs) and the plastic substrates which are routinely used for cell cultures. As a proof-of-concept we report the density-controlled synthesis of ZnO nanowires on flexible PCBs, thus opening the way to assembling compact intracellular-analysis systems, including nanowires, electronics, and microfluidics, on a single substrate.

The accurate control of the densities and dimensions of nanowires is often crucial. As an example, when nanowires are used to interface with cells, the intracellular access and the cell adhesion, morphology, viability, mobility and proliferation are all highly dependent on the dimensions and the densities of the nanowires^1^. In particular, long and high-density nanowires (*e.g.* more than 100 nanowires/100 μm^2^) can prevent the cell from making contact with the substrate and may limit the penetration through adhesion mechanisms^2^. For instance, by using nanowires with densities as low as 5 nanowires/100 μm^2^, Xu *et al.* have quantified the penetration of nanowires into the cellular membrane by observing dynamic ion delivery^3^; as another example, Na *et al.* monitored the endogenous enzymatic activity in living cells^4^ by means of low-density nanowires. In fact, the ability to control the geometrical parameters of the nanostructures is even more important than the cytotoxicity of the substrate which, eventually, can be easily reduced by coating with bio-compatible polymers that can also facilitate the cell adhesion through electrostatic or weak intermolecular interactions^5^. Nevertheless, the synthesis of nanowires with accurately controlled densities and dimensions is still challenging, especially for the very low densities (*e.g.* 1 to 10 nanowires/100 μm^2^) required for intra-cellular studies^2^. The problem is obviously exacerbated in case of low-cost plastic substrates which can only withstand low-temperature processes such as wet-chemical procedures. In particular, we suggest that, since microfluidic devices can be integrated on printed circuit boards (PCB)^6^, a compact lab-on-PCB for intra-cellular analysis, comprising electronics, microfluidics and nanowires, can in principle be fabricated on a single PCB; however, though nanowires on conventional PCBs have already been reported^7^,^8^,^9^ a critical task for fabricating such a system would be the density-controlled solution-growth of nanowires at the low density levels required for intracellular analyses.

A classic approach for controlling the density of quasi-1D nanostructures is to take advantage of patterning for defining the dimensions and the positions of the nanostructures and of catalysis for promoting the growth^1^. In fact, lithography allows the definition of seeds with arbitrarily low density^10^,^11^,^12^,^13^; however, taking into account the lateral growth of nanowires during the synthesis^14^, defining seeds with the required deep sub-micrometer size would generally be impossible with conventional optical lithography and may require electron beam lithography which, however, is an expensive and time-consuming process. Therefore, the direct deposition of catalytic nanoparticles can often be preferable. In particular, gold can effectively catalyse the growth of ZnO nanostructures, also due to its small lattice mismatch which, in the case of ZnO (001) and Au (111), is about 12.7%^15^ or, in the case of ZnO (101) and Au (111), is about 5%^16^. In fact, this approach has been widely reported for vapor-liquid-solid (VLS) processes. For instance, an accurate control of the density of ZnO nanowires has been achieved using Au-PMMA nanoparticles as catalysts^17^, resulting in nanowires densities ranging from about 25 nanowires/100 μm^2^ to about 1500 nanowires/100 μm^2^; moreover, the density could be further reduced by lowering the concentration of Au-PMMA nanoparticles, thus allowing to achieve the low densities required for intracellular studies^2^. However, VLS processes typically require very high temperatures (*e.g.* more than 700 °C) and are therefore incompatible with many low-cost substrates, including the plastic substrates (glass transition temperatures lower than 110 °C) which are routinely used for cell cultures and PCBs. Gold nanoclusters have also been applied to the wet-chemistry synthesis of ZnO nanostructures; for instance, 6–8 nm sized gold nanoclusters were inglobated into ZnO pyramidal structures with 15 nm to 25 nm side-edge length, with the gold nanoparticle seed located centrally at the basal surface^16^. However, there are very few examples of wet-chemical growth of epitaxial ZnO nanowires using Au nanoparticles and even the catalytic role of gold in such processes has not yet been fully investigated and understood. In fact, it has been suggested that gold film layers do not have catalytic properties in wet-chemical syntheses, but only epitaxially contribute to the ZnO nanowires growth^18^ by acting as an intermediate lattice-adaptation layer^19^. On the other hand, Xu *et al.*^20^ concluded that Au is catalytic because the presence of Au films results in better ZnO nanostructures and also because, in the case of substrates patterned with Au nano-lines or Au nano-dots, the amount of ZnO nanostructures obtained are related to the pattern densities. However, Chopra *et al.*^21^ proposed that Au nanoparticles did not have any catalytic property in their wet-chemical syntheses and that Au nanoparticles could only favour the nucleation of ZnO microwires due to good lattice match.

Here, unlike most reports aimed at maximizing the nanowires densities^19^,^22^,^23^,^24^, our goal is to grow density-controlled nanowires with the low densities required for penetrating the cellular membrane^2^ (*i.e.* approximately 1 to 10 nanowires/100 μm^2^). Therefore, first, we deposit a thin chromium film in order to suppress the ZnO nucleation^12^ down to negligible densities (*e.g.* 0.3 nanowires/100 μm^2^ or lower) and, then, we demonstrate that size selected gold nanoclusters (Au SSNCs) can effectively catalyse the ZnO nanowires growth in standard equimolar zinc nitrate-HMTA nutrient solutions. Remarkably, since, for a given number of nanoparticles, smaller particles resulted in higher densities of ZnO nanowires, we conclude that, first, the enhanced ZnO hetero-nucleation is dominated by catalysis rather than by layer-adaptation and, second, that the catalytic properties of the nanoparticles are stronger when shrinking the particle size, as expected. Among other factors, such as SSNC aggregation or atomic structure variations, a general argument to explain the observed trend would be that the surface to volume ratio increases with downscaling, so the smaller the nanocluster, the larger the fraction of superficial atoms^25^ and, therefore, the greater the expected catalytic activity^26^. Our approach is ideal for investigating the catalytic properties of gold as the nanoclusters are deposited by a cluster beam source that permits the direct transfer of highly pure Au nanoclusters with a totally free surface (no micelles, ligands or surfactants on the surface) and with accurately controlled size (*i.e.* 923 ± 22, 923 ± 20, 309 ± 7 and 147 ± 4 atoms), average coverage and kinetic energy during deposition^27^,^28^,^29^. The entire process does not involve any high temperature or annealing step. Though our method is general, as a proof-of-concept we have used conventional flexible PCB-substrates which would, of course, be the ultimate low-cost solution for assembling into a single substrate a complete intracellular analysis system comprising nanowires, electronics, and microfluidics.

## Materials and Methods

**Figure 1:**
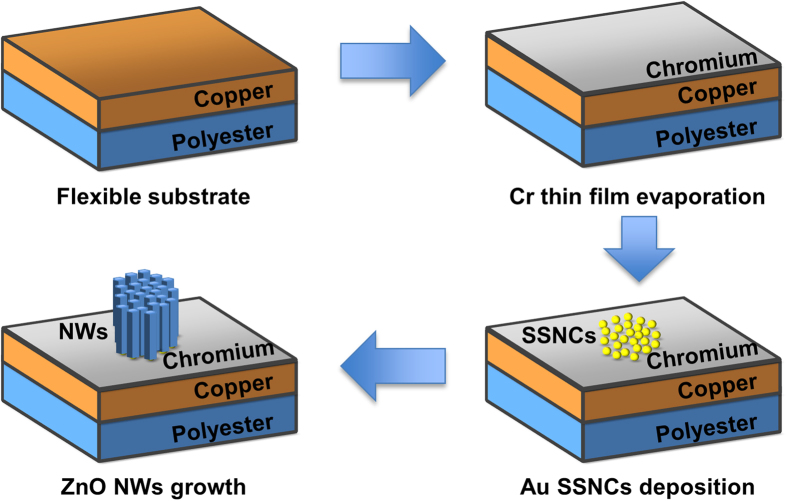
Schematic representation (not to scale) of the process for growing density-controlled ZnO nanowires on generic substrates (as a relevant example, a conventional printed circuit board, PCB, is used as a substrate).

Figure 1 shows a schematic representation of the fabrication process, namely chromium deposition, deposition of Au size-selected nanoparticles and solution growth of ZnO nanowires. As low-cost substrates we used flexible copper PCBs (Mega Electronics, Cambridge, UK; Supplementary Fig. S1 shows a long stripe of PCB before processing). The PCBs consist of a 50 μm thick polyester film, single side epoxy-bonded with an electro-deposited high-ductility (EDHD) 35 μm copper film. In order to inhibit the nucleation of ZnO nanowires down to negligible levels, we thermally evaporated an ~85 nm thick chromium^12^ layer on top of the copper layer of the PCB. Afterwards, we deposited size-selected gold nanoclusters onto the chromium layer using a magnetron-sputtering, gas-condensation cluster beam source^30^,^31^. The cluster source incorporates a time-of-flight mass filter^32^ which enables the accurate size selection of the nanoclusters prior to deposition in high vacuum (<10^−6^ mbar). The exit aperture allows control over the mass resolution of the filter. According to calibration with a beam of Ar^+^ ions, the nominal mass resolutions employed were M/∆M ≈ 21 (related to 10^9^ SSNCs/mm^2^ average density) and M/∆M ≈ 23 (related to 10^7^ SSNCs/mm^2^ average density), thus yielding nanoclusters that consist of 923 ± 22, 923 ± 20, 309 ± 7 and 147 ± 4 Au atoms. On the basis of our theoretical models, we estimate the cluster diameters averaged over the commonly-occurring high symmetry isomers (icosahedral, decahedral and face-centred cubic) as being 3.2 nm for Au_923_ (in close agreement with experiments^33^), 2.1 nm for Au_309_ and 1.6 nm for Au_147_. The SSNCs were deposited at energies of 1.6 eV/atom for Au_923±22_ and 1 eV/atom otherwise, which is considered to be close to the ‘soft-landing’ regime^27^,^34^. The nanoclusters are focussed through a 4 mm circular aperture prior to deposition onto the substrate. Since the beam current of size-selected nanoclusters is monitored at the substrate continuoulsy during deposition, the average density of the deposited nanoclusters can be determined by calculating the total dose (beam current integrated with respect to time) over the deposition area of the substrate (12.6 mm^2^).

After the deposition of the Au SSNCs, we placed the substrate upside-down floating on the surface of a water-based nutrient solution, composed by ultra-pure DI water (Barnstead Easypure II filtration system, 18.2 MΩ · cm) in which we dissolved equimolar concentrations (5 mM) of zinc nitrate hexahydrate (purum p.a., crystallized, ≥99.0% (KT)) and Hexamethylenetetramine (HMTA, ACS reagent, ≥99.0%) purchased from Sigma Aldrich. In order to promote the ZnO nanowires growth, we kept the Erlenmeyer flask containing the sample and the solution in a Memmert temperature controlled oven for 12 hours at 70 °C. We performed pH measurements with an ION 2700 Eutech Instrument equipped with an automatic system for temperature compensation. Following growth, we acquired scanning electron microscope (SEM) images of the samples with an FE-SEM (LEO SUPRA 1250, Oberkochen, Germany). In order to prevent charging of the sample we connected a corner of the metal layer of the PCB to the grounded SEM chamber, so that we could get high-quality images without covering the nanowires with a thin gold layer. Using the same conditions, we performed energy dispersive X-ray spectroscopy (EDX) with a Quanta INCA system. Besides different kinds of Au SSNCs, for the sake of comparison, we have also used control substrates without Au SSNCs.

## Results

The growth of ZnO in an equimolar zinc nitrate – HMTA solution, as discussed in detail by Zainelabdin *et al.*^35^, involves the reactions
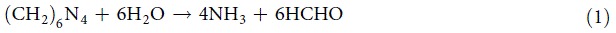

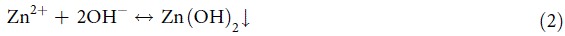

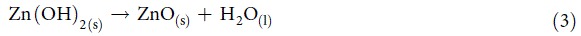


A typical pH measurement taken during these reactions is shown in Supplementary Fig. S2. The pH, initially at a value around 7.6, after some seconds, reaches 7 and then, during the initial solution heating, increases. Since water ionization is endothermic we observed that the pH increases with temperature^36^ up to 7.1 and then, after about 2 minutes, decreases down to about 6.8 after about 25 minutes. During these 25 minutes the solution becomes slightly turbid due to the formation of ZnO induced by the thermal decomposition of hexamethylenetetramine which produces OH^-^ groups (reaction 1), resulting in the production of thermodynamically unstable zinc hydroxide (reaction 2), which spontaneously gives rise to ZnO^37^ (reaction 3). After about 25 minutes the solution temperature is optimal (>60 °C) for HMTA decomposition and, therefore, the ZnO growth is more sustained so that the pH curve goes down with higher slope, until it reaches a final plateau around 6.3 after about 50 minutes. After the pH stabilizes and the regime temperature has been reached (70 °C in our case) continuous ZnO growth occurs throughout the 12 hours duration of the reaction. The measured pH of the nutrient solution, in the range 6.3 to 7.6 during the entire solution-growth process, is not aggressive towards chromium and thus is suited for the solution growth of ZnO nanowires onto the SSNC-functionalized chromium film.

**Figure 2:**
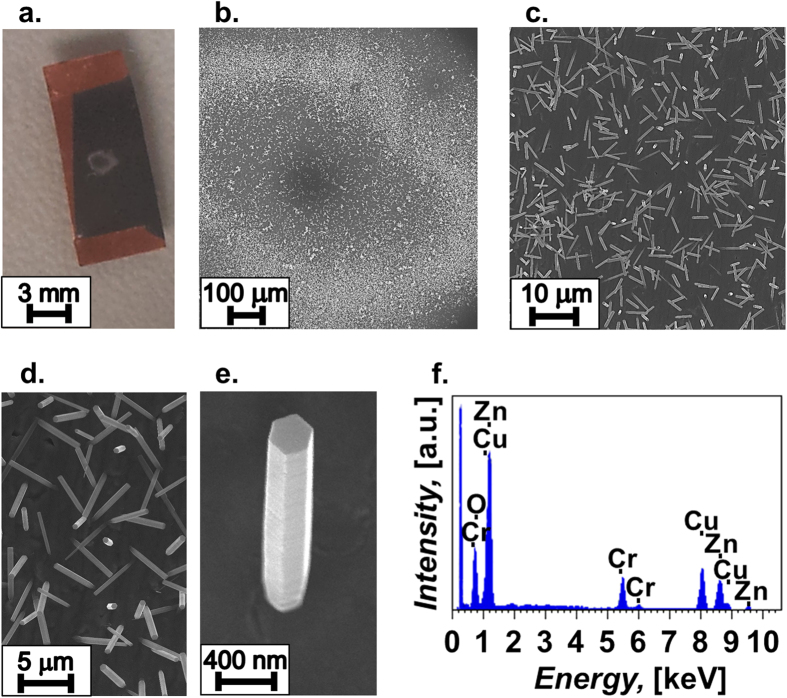
(**a**–**e**) Images of ZnO nanowires grown on a flexible PCB coated with a chromium thin film for inhibition and, then, by size-selected Au_923_ nanoclusters (SSNCs) for catalysis, with the SSNCs’ deposition energy at 1.6 eV/atom. (**a**) Photograph of the sample; the white region at the centre clearly reveals the growth of ZnO nanowires. (**b**) SEM image of the central region showing the growth in a ring-like pattern over the region where the clusters were deposited. (**c**) SEM image of the ring revealing a quite uniform distribution of the nanowires within the ring. (**d**) SEM image showing a few ZnO nanowires within the ring. (**e**) SEM image of a single ZnO nanowire within the ring. (**f**) EDX spectroscopy with peaks related to chromium, copper, zinc and oxygen.

**Figure 3:**
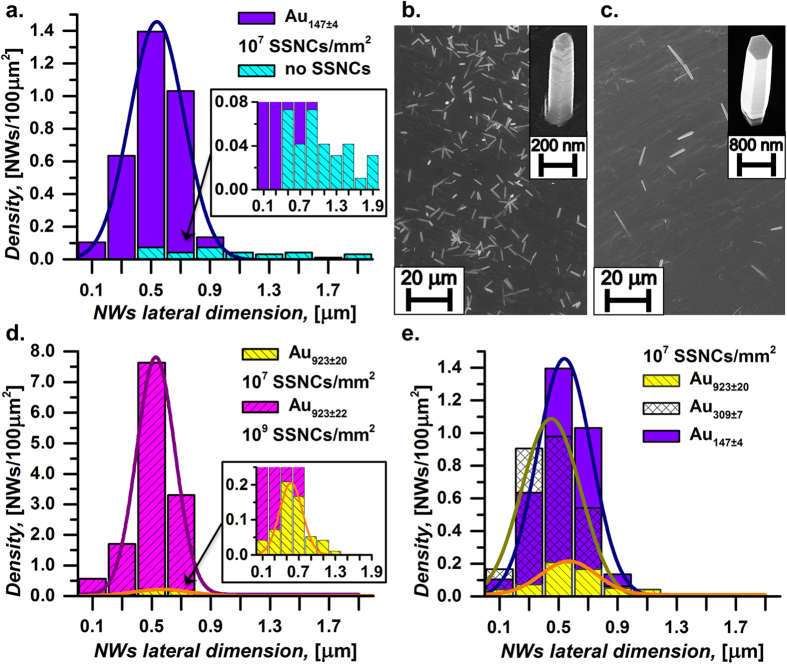
(**a**–**c**) Histograms and corresponding SEM images, showing a comparison of the size distributions of ZnO nanowires grown on Cr films with (**b**) and without (**c**) functionalization using size-selected nanoclusters (Au_147_ clusters deposited at an energy of 1.0 eV/atom); the insets show typical nanowires at higher magnification for both cases. (**d**) A comparison of nanowire densities for different densities of SSNC (10^9^ SSNCs/mm^2^ and 10^7^ SSNCs/mm^2^). (**e**) A comparison of nanowire densities for SSNCs of different sizes.

Figure 2(a) shows a photograph of a Cu-PCB substrate after all the processing steps illustrated in Fig. 1. The white central area in Fig. 2(a), with external ring diameter around 2 mm, is due to the growth of ZnO nanostructures on the part of the substrate which was pre-coated with Au_923_ clusters (focussed through an aperture with a diameter of 4 mm during deposition), thus confirming the catalytic activity of the SSNCs; the dark portion is the chromium layer; the orange corners are the parts of the PCB copper layer which were protected during the chromium evaporation. The growth of nanowires on the part of the Cr layer which was not covered by the gold SSNCs is negligible, thus confirming the inhibiting role of chromium. The presence of the Au nanoparticles effectively increases the number of nucleation sites. As evident from Fig. 2(b) the growth is maximum in an annular, ring-like, region, which we attribute to the variation in density of clusters deposited on the substrate. Indeed, the profile of a highly focussed cluster beam can follow a non-uniform distribution, and therefore the flux of nanoclusters can decrease radially from the centre of the beam^31^. Scanning probe microscopy studies of clusters are typically performed on (near-)atomically flat substrates and are not suitable for the accurate characterization of the local nanocluster density on our low-cost substrates (flexible PCBs). However, from HAADF-STEM images of Au_923_ nanoclusters produced using a highly focussed cluster beam and then deposited onto amorphous carbon films, we estimated that the nanocluster density reduces to around one-third at a distance of 0.8 mm from the centre. Consequently, we may consider that the local density of nanoclusters on the Cr-coated PCB substrates should also follow the same cluster beam profile, with the density falling off towards the edge of the beam. In the centre where the density of nanoclusters is high, short-range diffusion can lead to aggregation; further away from the central area, there would be a higher density of unaggregated clusters. It seems likely therefore that the ZnO nanowires grow in a ring-like pattern because preferential nucleation takes place in the region where the nanoclusters are less aggregated, consistent with the stronger catalytic properties of smaller nanoparticles (see Fig. 3 and discussion). In the present work, variations in the local density of as-deposited nanoclusters are likely to arise due to the use of a highly focussed cluster beam during deposition. In future, more uniform nanocluster coverages can be achieved by selecting (with an aperture) the central region of a less focussed (more diffuse) cluster beam prior to deposition. Additionally, the process of rastering allows deposition of nanoclusters over larger areas (e.g. ~cm^2^), whereby the substrate is moved in front of the beam in an automated fashion, effectively ‘printing’ the nanoclusters line-by-line in order to build up an even coverage across the substrate.

The SEM images in Fig. 2(c–e) confirm the growth of low-density ZnO nanowires and shows the regular hexagonal shape of the ZnO nanowires due to their hexagonal wurtzite structure^38^; a typical XRD θ–2θ spectrum (Co K_α_ radiation) performed on the nanowires is shown in Supplementary Fig. S3 and validates the crystallinity of the nanowires. The diameters of the nanowires range in the hundreds of nanometers, which is obviously much wider than the single Au cluster because of the lateral growth following the initial nucleation, as common in the solution growth of nanowires^14^. Fig. 2(f) reports the EDX spectroscopy of the nanowires and reveals the presence of chromium, copper, zinc and oxygen. Remarkably, due to the ultra-small amount of gold deposited and due to the possible incorporation inside the nanowires, we could not detect any presence of gold.

Figure 3 shows that both the size and the density of the deposited Au SSNCs heavily affect both the densities and the dimensions of the nanowires grown on the Cr surface (identical conditions, for all the samples, with reference to nutrient solution, growth time, and temperature, see the Materials and Methods section). As to the statistical analyses, we have used the highest-density regions with areas of 9600μm^2^ for the low-density samples (*e.g.* for the control samples) or of 878μm^2^ for the high density samples. For completeness, we provide the numerical data on the nanowires densities for the different samples in Supplementary Table ST1. For graphical clarity, in Fig. 3(a,d,e), besides the histograms, we also show the Gaussian fits to the collected data. As shown in Fig. 3(a), even few SSNCs, about 10^7^ Au_147±4_ SSNCs/mm^2^ can substantially improve the nucleation of ZnO nanowires on the Cr surface. Differently from the flat surface case^12^, the nucleation of ZnO nanowires on the Cr film deposited on the flexible PCBs occurs even in absence of Au SSNCs (control samples) and is likely ascribed to singular morphological defects, cavities and hollow zones of the surface of the low-cost flexible PCB, as such irregularities can significantly lower the activation energy for the nucleation of ZnO^39^,^40^,^41^. However, in these control samples, we observed very few nanowires exhibiting very irregular diameters, with a broad size distribution in the range from 400 nm to 2 μm. By contrast, in the samples where the chromium film has been functionalized with the Au SSNCs we found much higher nanowires densities, with a narrow statistical distribution in size, and a mean diameter on the order of 500 nm. Fig. 3(b) shows a typical SEM image of the central area of a processed sample which, before the wet-chemical synthesis of the nanowires, has been functionalized with Au SSNCs; as evident, the nanowire density is in the range of interest for cell analysis and the nanowires are relatively uniform. For comparison, Fig. 3(c) reports a typical SEM picture of the central area of samples without Au SSNCs, with the zoom of a single nanowire in the inset; in this case, there are very few nanowires, with larger and very broadly distributed diameters (as expected, as these nanowires grow on chromium because of the irregularities of the underlying PCB). By contrast, the deposition of Au SSNCs results in both an increased number of nucleation sites and much more uniform nanowires (as expected due to the accurately controlled size of Au SSNCs). We have also used more dense distributions of Au SSNCs; in particular, Fig. 3(d) compares samples irradiated with Au_923_ nanoclusters by using different SSNCs densities equal to 10^9^ SSNCs/mm^2^ and 10^7^ SSNCs/mm^2^, respectively. In both these cases the dose and deposition energies were similar (between 1.0 eV/atom and 1.6 eV/atom) and related to the soft-landing regime. The densities of the solution-grown nanowires were significantly different with 13 nanowires/100μm^2^ for 10^9^ SSNCs/mm^2^ and of 0.59 nanowires/100 μm^2^ for 10^7^ SSNCs/mm^2^ while the mean lateral dimensions were similar with values of 530 ± 130 nm for 10^9^ SSNCs/mm^2^ and of 570 ± 190 nm for 10^7^ SSNCs/mm^2^, as shown in Fig. 3(d). We also found that using nanoclusters of different sizes also affects the density of the ZnO nanowires. In fact, Fig. 3(e) shows the nanowire distributions for samples functionalized with Au SSNCs of comparable average densities (10^7^ SSNCs/mm^2^) and deposition energy (1.0 eV/atom), but with different sizes (Au_923±20_, Au_309±7_, Au_147±4_). Most remarkably, as evident in Fig. 3(e), the maximum density of nanowires is found on the sample functionalized with the smallest particles (Au_147±4_), thus providing good evidence that the enhanced nucleation is dominated by heterogeneous catalysis rather than by lattice adaptation (which would likely be minimum on the sample containing the smallest gold particles). This is also consistent with the expected stronger catalytic activity of smaller Au nanoclusters and in agreement with previous studies on gold catalysis^26^,^42^,^43^ which have found that both size and shape of the Au nanoclusters are crucial parameters for the enhancement of chemical reactions. We also mention that, though here we focused on the most widely used wet-chemical recipe for growing ZnO nanowires, changing the conditions of the chemical reaction may offer additional opportunities to control the key nanowires parameters.

In conclusion, the growth of nanowires with the low density levels required for intracellular analyses is very challenging, especially in case of low-cost substrates which may not withstand high temperatures. Here we have demonstrated that by depositing, first, a thin chromium film as an inhibitor for the ZnO nucleation and, then, size selected gold nanoclusters (Au SSNCs) as catalysts, it is possible to control the density of the solution grown ZnO nanowires; the entire process does not involve any high temperature step and allows the growth of ZnO nanowires at the low densities which are, in principle, suitable for cellular membrane penetration and for subsequent cells analysis. Remarkably, by depositing pure Au nanoclusters without any micelles or additives, which could otherwise induce some sort of contamination, we found that, first, gold nanoclusters can greatly enhance the in-solution nucleation of ZnO nanowires primarily because of catalysis rather than lattice adaptation and, second, the smaller nanoclusters are more effective catalysts. The density of the ZnO nanowires can be readily tuned by adjusting the density and the size of the deposited Au nanoclusters; moreover, the diameters of the ZnO nanowires are reduced and are much more uniform when compared to the case without Au SSNCs. Finally, as a proof-of-concept we have demonstrated the fabrication of ZnO nanowires with densities compatible with intracellular analyses on conventional, low-cost flexible printed circuit boards (PCBs). Though the proposed method could find many applications, our results can, in particular, be an important step towards the fabrication of low-cost and compact intracellular-analysis systems including nanowires, electronics, and microfluidics on a single substrate.

## Additional Information

**How to cite this article**: Errico, V. *et al.* Chromium inhibition and size-selected Au nanocluster catalysis for the solution growth of low-density ZnO nanowires. *Sci. Rep.* **5**, 12336; doi: 10.1038/srep12336 (2015).

## Supplementary Material

Supplementary Information
