# Supplementary material for: Chromium inhibition and size-selected Au nanocluster catalysis for the solution growth of low-density ZnO nanowires
Source: Sci Rep. 2015 Jul 23;5:12336. doi: 10.1038/srep12336 (PMC4511950; doi:10.1038/srep12336)
Supplement: Supplementary Information [file srep12336-s1.pdf]

Chromium inhibition and size-selected Au nanocluster catalysis for the solution growth of low-density ZnO nanowires

Vito Errico [1], Giuseppe Arrabito [1], Simon R. Plant [2], Pier Gianni Medaglia [3], Richard E. Palmer [2 §], Christian Falconi [1 §]

[1] Department of Electronic Engineering, University of Rome Tor Vergata, Via del Politecnico 1, 00133, Rome, Italy

[2] Nanoscale Physics Research Laboratory, School of Physics and Astronomy, University of Birmingham, Edgbaston, Birmingham, B15 2TT, United Kingdom

[3] Department of Industrial Engineering, University of Tor Vergata, Via del Politecnico 1, 00133, Rome, Italy

[§] Corresponding authors: r.e.palmer@bham.ac.uk, falconi@eln.uniroma2.it

SUPPLEMENTARY INFORMATION

Flexible PCB and pre-processing procedure

Supplementary Fig. S1 shows the low-cost flexible PCB we have used for the experiments; the PCB stripe is bent in order to emphasize its high flexibility. Before processing, we cut the PCB in samples with dimension of 10mm x 5mm, we removed the resist with acetone (Carlo Erba, RPE-ACS-ISO, ≥99.8%) and subsequently extensively rinsed with ethanol (Sigma Aldrich, puriss.p.a., ACS reagent, ≥99.8%) and Millipore water (Millipore milli-Q, 18.2 MΩ·cm).

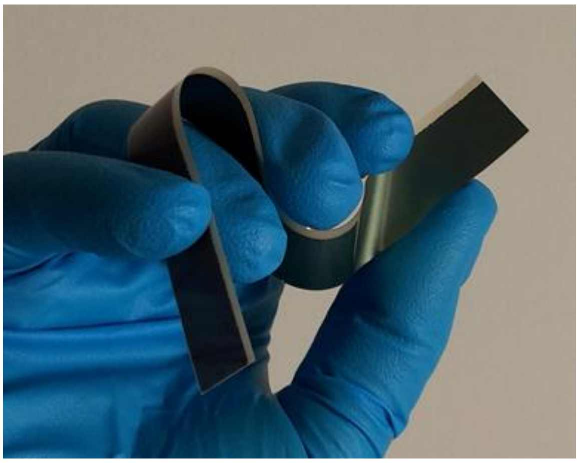

Figure S1: Photograph of a non-processed flexible PCB.

pH during the growth of ZnO nanowires

During the ZnO nanowire growth (in almost neutral pH conditions) by the aqueous $Zn(NO_3)_2$-HMTA chemistry, hexamethylenetetramine (HMTA, Sigma Aldrich, ACS reagent, ≥99.0%) acts as a pH buffer through its slow decomposition to formaldehyde and ammonia. Ammonia reacts with water and slowly produces NH_4^+ and OH^-. NH_4^+ forms a complex with the zinc ions as a buffering mechanism. Importantly, the slow release of OH^- groups by HMTA permits sustained nucleation and continuous growth of ZnO nanowires at pH values that are not aggressive towards chromium. We performed in-situ continuous measurements of pH during ZnO nanowire synthesis and in Supplementary Fig. S2 we report a typical pH measurement, which provides evidence of such pH buffering: after 50 minutes the pH stabilizes near 6.3, as expected, until the end of the process.

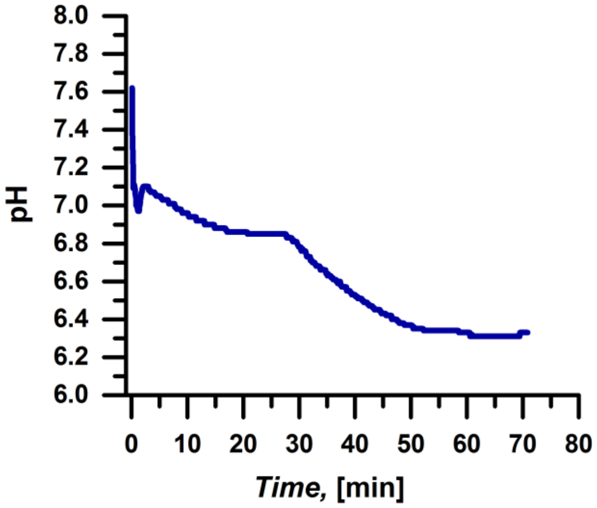

Figure S2: Typical measurement of pH as a function of time for an aqueous solution containing 5 mM $ZnNO_3{\cdot}6H_2O$ and 5 mM HMTA during the growth carried out at 70°C on a SSNC-chromium covered copper PCB substrate.

XRD measurements

We performed XRD measurements on ZnO nanowires grown on samples functionalised with Au SSNCs by a Rigaku Geigerflex Bragg Brentano θ-2θ diffractometer (Co K_α radiation) and in Supplementary Fig. S3 we report a typical acquired spectrum. Due to the extremely low density of ZnO nanowires, we achieved peaks evidence only after several hours of counting integration and focusing the analysis on the angular range 35°-45°, where typically wurtzite-type structures exhibit the most intense reflections. Despite of a noisy background, the acquired XRD spectrum shows three main peaks associated to differently orientated nanowires, thus confirming the good degree of crystallinity and the hexagonal wurtzite-type structure. Furthermore, from the angular positions of (100), (002) and (101) reflections we could evaluate the *c* and *a* lattice parameters, using the following relationship, well known for hexagonal structures:

$$\frac{1}{d^2} = \frac{4}{3}\left(\frac{h^2 + hk + k^2}{a^2}\right) + \frac{l^2}{c^2},$$

where *d* is the distance between adiacent planes in the (hkl) set. The values obtained, (*c*=5.21±0.02 Å, and *a*=3.22±0.02 Å) are in good agreement with the results reported in the literature for ZnO wurtzite-type crystal.

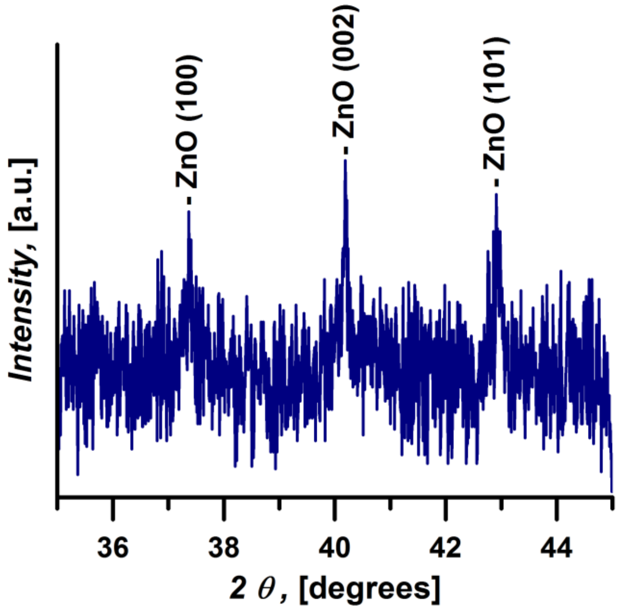

Figure S3: A typical XRD θ- 2θ spectrum (Co K_α radiation) performed on low-density ZnO nanowires grown on Cr film with Au SSNCs functionalization. Different reflection peaks are visible, thus confirming the nanowires crystallinity and their different orientations.

ZnO nanowire densities

Supplementary Table ST1 shows the ZnO NW densities measured from SEM pictures using the ImageJ software (Wayne Rasband National Institute of Health USA, http://imagej.nih.gov/ij).

As reported in the last line of the table (ratio between the SSNCs density and the NWs density), we estimated the mean number of clusters seeding the growth of each NW.

We can observe from samples with density of 10^7 SSNCs/mm^2 (reported in the latest three columns in the Supplementary Table ST1) that, due to the increased catalytic effect, the smaller is the SSNCs dimension the lower the number of SSNCs required for the growth of a single NW. Moreover, from Au_{923} SSNCs samples (2nd and 3rd columns in the Supplementary Table ST1), we observed a reduced catalysis effect associated to the greater SSNCs density, since the growth of a single NW required a mean of 7569 SSNCs for a density of 10^9 SSNCs/mm^2 compared to 1684 SSNCs involved for a density of 10^7 SSNCs/mm^2.

***Lateral dimension range* [μm]**	***Control sample No SSNCs* [NWs/100μm^2]**	***$Au_{923\pm22}$ 10^9 SSNCs/mm^2* [NWs/100μm^2]**	***$Au_{923\pm20}$ 10^7 SSNCs/mm^2* [NWs/100μm^2]**	***$Au_{309\pm7}$ 10^7 SSNCs/mm^2* [NWs/100μm^2]**	***$Au_{147\pm4}$ 10^7 SSNCs/mm^2* [NWs/100μm^2]**
**0 - 0.2**	-	0.57	0.042	0.17	0.10
**0.2 - 0.4**	-	1.7	0.073	0.91	0.64
**0.4 - 0.6**	0.073	7.6	0.21	0.98	1.4
**0.6 - 0.8**	0.042	3.3	0.17	0.54	1.0
**0.8 - 1.0**	0.073	-	0.052	0.063	0.14
**1.0 - 1.2**	0.042	-	0.042	-	0.021
**1.2 - 1.4**	0.031	-	0.010	-	-
**1.4 - 1.6**	0.042	-	-	-	-
**1.6 - 1.8**	0.010	-	-	-	-
**1.8 - 2.0**	0.031	-	-	-	-
**Total**	0.34	13	0.59	2.7	3.3
**SSNCs/NW**	-	7569	1684	376	301

Table ST1: numerical results of the statistical analysis (see Figure 3 in the main text).
